# Design and Fabrication of Extremely Lightweight Truss-Structured Metal Mirrors

**DOI:** 10.3390/ma15134562

**Published:** 2022-06-29

**Authors:** Chen Liu, Kai Xu, Yongqi Zhang, Haifei Hu, Xiaoping Tao, Zhiyu Zhang, Weijie Deng, Xuejun Zhang

**Affiliations:** 1 Key Laboratory of Optical System Advanced Manufacturing Technology, Changchun Institute of Optics, Fine Mechanics and Physics, Chinese Academy of Sciences, Changchun 130033, China; liuchen181@mails.ucas.ac.cn (C.L.); xukai201@mails.ucas.ac.cn (K.X.); huhf@ciomp.ac.cn (H.H.); taoxp@ciomp.ac.cn (X.T.); dengwj@ciomp.ac.cn (W.D.); zxj@ciomp.ac.cn (X.Z.); 2 University of Chinese Academy of Sciences, Beijing 100049, China; 3 Army Academy of Armored Forces, Changchun 130117, China; zhangyongqi0309@163.com

**Keywords:** lightweight mirror, additive manufacturing, finite element analysis, laser modification, optical fabrication

## Abstract

Three-dimensional printing, also called additive manufacturing (AM), offers a new vision for optical components in terms of weight reduction and strength improvement. A truss, which is a triangulated system of members that are structured and connected in such a way that they mainly bear axial force, is commonly used in steel structures to improve stiffness and reduce weight. Combining these two technologies, an extremely lightweight truss-structured mirror was proposed. First, the finite element analyses (FEA) on surface shape deviation and modal properties were carried out. Results showed that the mirrors had sufficient stiffness and a high weight reduction of up to 85%. In order to verify their performance, the truss-structured mirror blanks were fabricated with AM technology. After that, both the preprocessing and the postprocessing of the mirrors were carried out. The results show that without NiP coating, a surface shape deviation of 0.353λ (PV) and 0.028 λ (RMS) (λ = 632.8 nm) with a roughness of Ra 2.8 nm, could be achieved. Therefore, the truss-structured mirrors in this study have the characteristics of being extremely lightweight and having improved stiffness as well as strong temperature stability.

## 1. Introduction

Infrared optical telescopes in space are important tools for observing the formation of stars, studying the galaxy evolution, as well as finding the first galaxies born in the universe. In order to reduce launch costs, the primary mirror of an optical telescope is generally made of materials with high stiffness-to-density ratio, such as glass, ceramics, and lightweight but strong metals. For a specific type of material, it is very important to further reduce the mass of the mirror through lightweight design without sacrificing stiffness. For example, the James Webb Space Telescope (JWST) is the largest and most powerful infrared space telescope. JWST’s primary mirror adopts the lightweight material beryllium and an open-back, lightweight design to maintain extreme stability while being comparably lightweight [1].

The lightweight design of mirrors has experienced a process from the open-back type, semi-open-back type and closed-back type, realized by technologies such as: (1) casting, (2) mechanical machining, and (3) additive manufacturing. Casting is the most commonly used method of weight reduction for difficult-to-machine materials such as glass or ceramics. Hence, these kinds of mirrors are open-back or semi-open-back types. Obviously, a closed-back type is more desirable as its mechanical stability is higher than that of the former two types. 

With the advent of metal mirrors, it has been possible to reduce a large amount of weight by turning or milling, making it possible for the mirrors to have a completely closed back. Although this is beneficial to improve the stiffness, the machining of these mirrors is still limited due to the low accessibility of the interior material, resulting in a weight reduction of less than 50% [2]. Therefore, it is difficult to reduce the mirror weight without sacrificing its stiffness by traditional machining methods. 

However, 3D printing, also called additive manufacturing (AM), could break the above limits. AM is a fabrication process that builds an object layer-by-layer, which promotes complex structures that cannot be achieved by subtractive machining [3,4]. In particular, selective laser melting (SLM) is a well-developed high-efficiency AM technology for the fabrication of metal parts with complex geometries [5]. The unique capabilities of SLM provide great design freedom for designers to use cellular structures or lattice structures. For example, Heidler et al. fabricated a mirror with a closed back. Therein, the internal structure of the mirror body was similar to a foam, which had a 64% weight reduction [6]. Hilpert et al. fabricated a honeycomb-structured closed-back mirror using SLM technology. The weight reduction factor rose to 70% while maintaining high stiffness [2]. 

Compared with the above-mentioned mirror structures, the truss structure can further increase the weight reduction factor of mirrors. A truss is a triangulated system that is structured and connected in such a way that it mainly bears axial force. Truss structures possess the merits of long span, high stiffness and load capacity [7]. In engineering, trusses are often used to construct the steel structures of buildings and bridges due to their lightweight and excellent mechanical properties. In fact, trusses have also been widely used in telescopes. JWST’s folding and unfolding system is realized through four trusses designed as an expandable tripod bracket [8,9]. 

However, to our best knowledge, a truss-structured mirror has not been reported. In this study, we proposed truss-structured mirrors. Finite element analyses (FEA) on the surface shape deviation and modal properties were performed. The mirrors had sufficient stiffness with weight reduction factors up to 80%. AM technology was used to fabricate the mirror blanks. Both preprocessing and postprocessing of the truss-structured mirrors were carried out. The findings of this study provide insights into the applications of extremely lightweight mirrors. 

## 2. Design of the Truss-Structured Mirrors

### 2.1. Truss Cell

**Figure 1:**
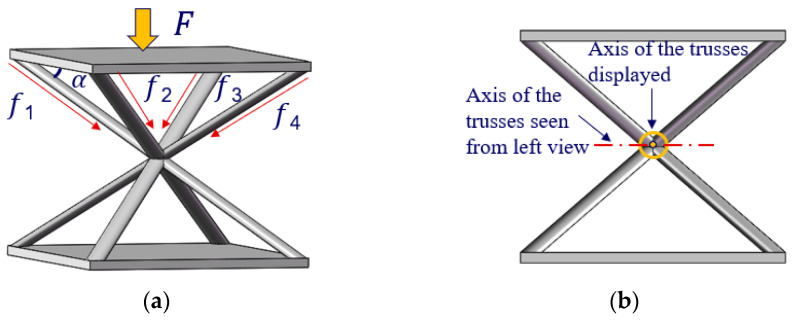
(**a**) Transmission of force load in a truss cell; (**b**) front view of the truss cell.

A cell of the truss structure as shown in Figure 1 was designed. Each rod is connected to one corner of the cell surfaces. Supposing the upper surface bears the force load *F*, it can be uniformly transmitted to the bottom surface through each rod with a force component of *f*. The relationships are expressed in Equation (1).

(1) $$f_{1}=f_{2}=f_{3}=f_{4}=\frac{F}{4\;\times \;\cos\alpha }$$

where α is the angle between each rod and the cell surface. For a truss cell, the maximum deformation *W* of the surface can be found using Equation (2).

(2) $$W\;=\;\Psi {\left[\frac{Et^{3}}{12(1-v^{2})}\right]}^{-1}Pb^{4}$$

where *E* is the Young’s modulus, *t* is the thickness of the mirror surface, *ν* is Poisson’s ratio, *P* is the pressure uniformly distributed on the mirror surface, *b* is the diameter of the inscribed circle within the square truss, *ψ* is a constant related to the shape and in our design the *ψ*_square_ = 0.00126 [10].

Moreover, the rod arrangement greatly reduces the weight of the truss cell, thereby improving the weight reduction factors. As shown in Figure 1b, the axis of the observed truss cell is vertical to the axes of the truss in adjacent planes. Thus, the proposed truss cell structure could facilitate the uniformity and stability of the combination of multiple truss cells. Most importantly, the high strength of each truss cell ensures high stiffness of the truss mirrors.

### 2.2. Truss Mirrors

A single-layer truss-structured mirror was obtained by the arrangement and combination of the truss cells. With regard to a truss-structured mirror, *b* is the length of the side parallel to the mirror and *P* is the pressure acting on each cell. From Equation (2), it could be concluded that if a smaller value of *W* is expected, the value of *b* should be smaller, which means that more truss cells are required. However, if there are too many truss cells in a mirror, the value of the weight reduction factor will be reduced. Therefore, on the premise of meeting design requirements, it is better to make the trusses sparser to reduce the mirror weight.

In this study, we designed truss-structured mirrors whose outer shape was 150 mm × 150 mm × 41 mm with a mirror surface thickness of 2 mm. AlSi_10_Mg is widely used in SLM and therefore was chosen as the mirror material. The maximum deformation *W* of the mirror surface was calculated using Equation (2). The main parameters used in the calculation are shown in Table 1. 

If there is only one truss cell, the value of *b* is obviously equal to the side length (150 mm) of the mirror. The calculated maximum deformation *W* of the mirror is 7089 nm. When the number of truss cells increases to 3 × 3, the value of *b* will reduce to 1/3 of the side length. Then, the maximum deformation *W* will decrease rapidly to 88 nm, because it is changed with the fourth power of *b*. While the number of truss cells increases to 5 × 5, the calculated maximum deformation *W* is only 11 nm, which fully shows that the truss deformation converges rapidly with the increase in the number of truss cells. In this study, because the truss-structured mirrors are expected to be used in near-infrared and even visible light optics, the maximum deformation *W* should be controlled within tens of nanometers. Therefore, the number of truss cells designed in this study was set to 5 × 5.

**Figure 2:**
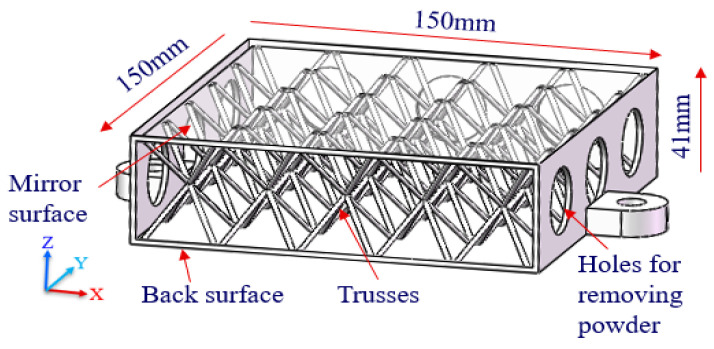
Cut view of the mirror model with single-layer truss structure.

**Figure 3:**
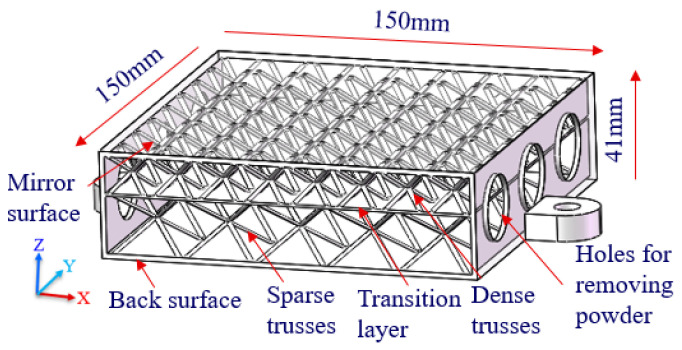
Cut view of the mirror model with double-layer truss structure.

The CAD model of a single-layer truss-structured mirror is shown in Figure 2. The truss cells symmetrically support the mirror surface and the back surface. Therefore, both surfaces have the same stiffness. In fact, the deformation control of the mirror surface should be more critical than that of the back one, and the processing of the mirror surface has higher requirements as well. In order to improve the stiffness of the mirror surface, a double-layered truss, as shown in Figure 3, was further studied. In this way, the truss cells directly supporting the mirror surface became dense while the underlying truss cells were set to be sparse. As a result, the mirror became more supportive, although the weight reduction factor was becoming somewhat lower. 

**Figure 4:**
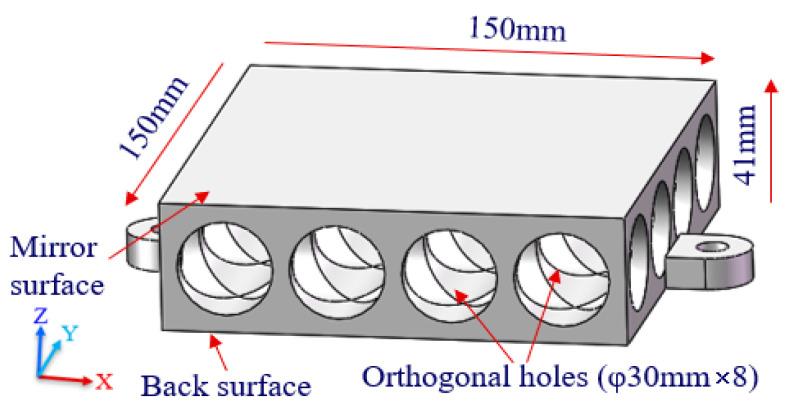
The traditional mirror model with orthogonal holes array.

In order to facilitate the comparative analysis, a traditional lightweight mirror with an orthogonal holes array was also designed. According to Equation (2), the deformation *W* is related to *b* under the same mirror surface thickness and material. As shown in Figure 4, the external dimensions were exactly the same as the truss-structured mirrors. The diameter of each hole, namely *b*, was set to 30 mm, which ensured that the deformation of the mirror with the orthogonal holes array was almost equal to that of the truss-structured mirrors. 

The three mirrors had the same designed deformation, namely, the same stiffness, but their weight reduction factors were significantly different. After calculation, the weight reduction factor for single-layer and double-layer truss-structured mirrors was 86.5% and 84.1%, respectively, while that of the traditional mirror with orthogonal holes array was only 61.6%. 

### 2.3. FEA Verifications

#### 2.3.1. Surface Shape Deformation

The material parameters used in the FEA model are listed in Table 1. Quadratic tetrahedral element property (Tet10) was applied to all free-type meshes for accuracy improvement. It is recognized that the denser the mesh, the more accurate the simulation, but this results in more computation time. In our previous work, a mesh size of ~2 mm was acceptable to achieve a computation error of less than 5% for a meter-level mirror structure [11]. Consequently, the mesh number for the aforementioned traditional, single-layer and double-layer truss-structured mirrors was 73,847, 279,933 and 486,369, respectively.

**Figure 5:**
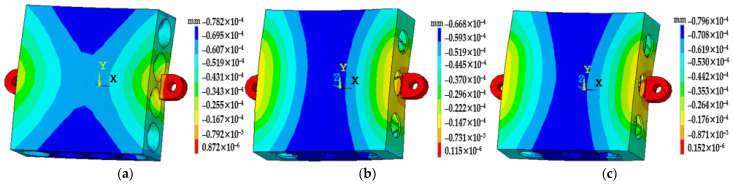
Surface deformations under gravity in Z direction: (**a**) traditional mirror; (**b**) single-layer truss mirror; (**c**) double-layer truss mirror.

**Figure 6:**
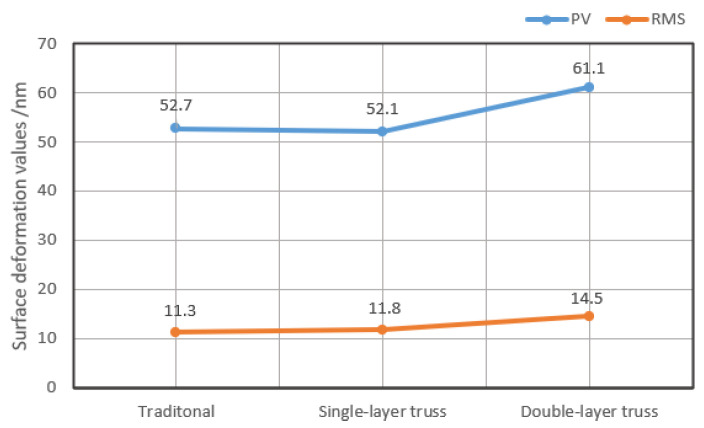
Surface deformation results in Figure 5.

The FEA verifications of the above three mirrors were carried out in a load case where the double mounting ears were fixed. The deformation contours of the mirror surfaces under 1G (G = 9.8 m/s^2^) acceleration gravity load in Z direction are shown in Figure 5. The node deformation data of the mirror surfaces were extracted in Figure 6. The statistical PV and RMS values of the traditional mirror and single-layer truss mirror were roughly the same, while those of the double-layer truss mirror were about 17% larger, indicating that the double-layer truss mirror had a relatively weak stiffness to resist gravity deformation. 

**Figure 7:**
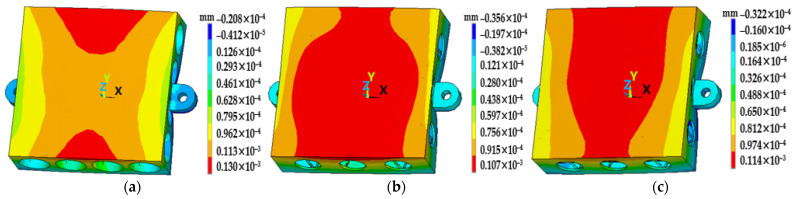
Surface deformations under 4 °C temperature rise: (**a**) traditional mirror; (**b**) single-layer truss mirror; (**c**) double-layer truss mirror.

**Figure 8:**
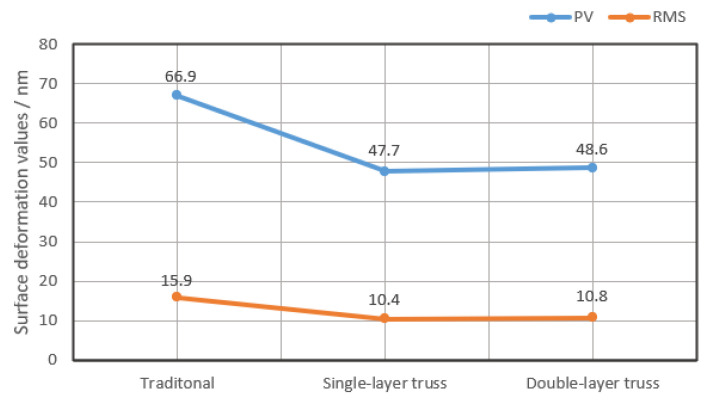
Surface deformation results in Figure 7.

Next, the FEA verifications of the above three mirrors were carried out in a load case of a temperature rise of 4 °C according to the application requirements. The deformation contours of the mirror surfaces are shown in Figure 7. The node deformation data of the mirror surfaces were also extracted and are shown in Figure 8. The PV and RMS values of the single-layer truss mirror and the double-layer truss mirror were almost the same, and much better than that of the traditional mirror. That is probably because the mass of the former mirrors was more evenly distributed, the mirror blanks could expand evenly under differing temperature loads. This is reflected in the smaller deformations in Figure 8.

#### 2.3.2. Modal Analysis

**Figure 9:**
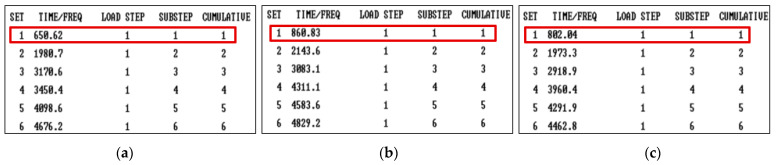
The natural frequency values: (**a**) traditional mirror; (**b**) single-layer truss mirror; (**c**) double-layer truss mirror.

Modal analysis is a common method used to study the dynamic characteristics of structures. As for the mirrors, it is critical to enhance the first-order natural frequency value to improve their shape stability under the conditions of complex vibration load. Figure 9 shows the results of modal analysis through FEA. The vibration direction was along the line connecting the two mounting ears. The first-order natural frequency values of the traditional mirror, the single-layer truss mirror and the double-layer truss mirror were 650 Hz, 861 Hz and 802 Hz, respectively. Therefore, the truss-structured mirrors had better dynamic performance compared with the traditional one. 

## 3. Fabrication of Truss-Structured Mirrors

### 3.1. Mirror Blanks Fabrication

**Figure 10:**
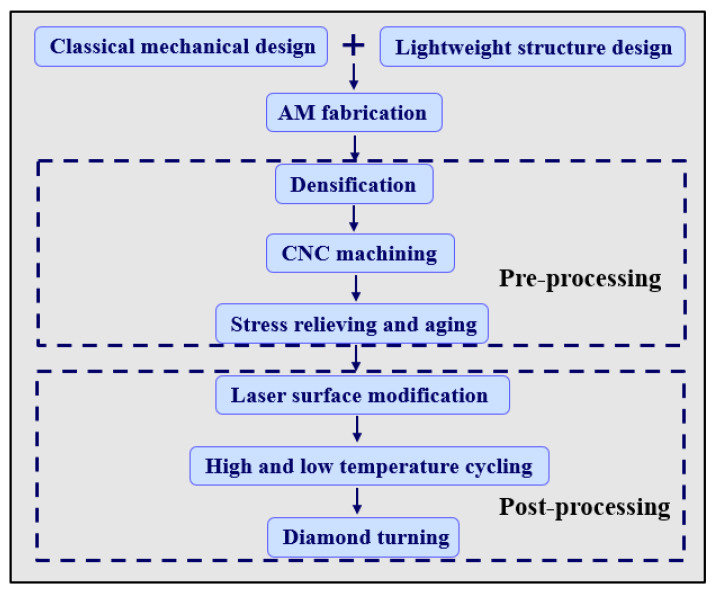
Processing chain to generate the truss-structured mirrors.

The processing chain for the mirrors is shown in Figure 10. In this study, SLM technology was used to fabricate the truss-structured mirrors. The material of the mirror blank was gas-atomized AlSi_10_Mg powder supplied by BAM LTD. All specimens were fabricated using an FS271M system (laser-powder-bed fusion) with Yb-fiber laser. The forming process was carried out in an argon atmosphere with an oxygen mass fraction of less than 0.1%. The SLM processes were performed with a laser power of 370 W, scan speed of 1300 mm/s and hatch distance of 80 μm.

### 3.2. Preprocessing for Mirror Blank Stabilization

During the SLM processing, AlSi_10_Mg powder particles were not completely melted, which would cause the formation of void defects inside the mirror bodies. Therefore, a densification treatment was required to eliminate macroscopic and microscopic void defects. Hot isostatic pressing (HIP) [12] was used to reduce the porosity of AMed workpieces. In HIP treatment, the workpieces were placed in a heat treatment furnace at a temperature of ~510 °C and a pressure of ~110 MPa for about 2 h. Finally, the workpieces were cooled with the furnace. 

After densification, semi-finishing of the mirror surface was carried out. Computer numerical control machining (CNC) was used to machine the surface roughness (Ra) to about 1.6 μm. It is known that the aging treatment is a process to improve mechanical properties and reduce the residual stress of workpieces [13]. Therefore, the aging treatment was applied at the temperature of 130 °C with a dwell time of 4 h [14]. 

### 3.3. Postprocessing for Surface Modification

**Figure 11:**
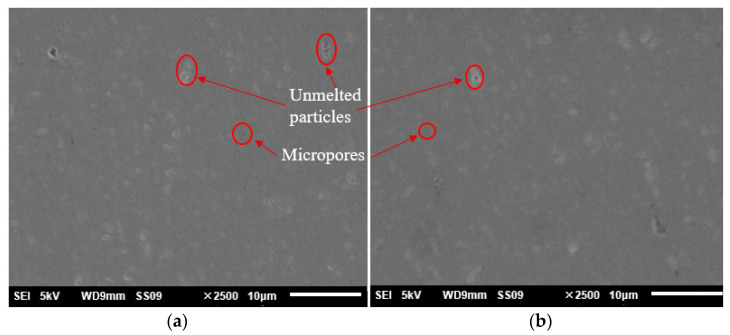
SEM micrograph of the mirror surfaces: (**a**) result at first point; (**b**) result at second point.

As shown by the red circle in Figure 11, there were unmolten particles and micropores on the mirror surfaces, which not only degraded the surface quality but also reduced the surface densification. It is known that the mirrors used in visible and infrared systems have to be plated with a NiP layer to increase the surface roughness, but this may lead to bimetal effects because of the different thermal coefficients between NiP and the mirror blanks [15].

In postprocessing, we proposed to modify the surface quality by pulse laser irradiation [16]. A semiconductor nanosecond laser was used to modify the surface conditions. The laser has a wavelength of 532.5 nm with a spot diameter of about 80 μm. During laser irradiation, laser energy density has a remarkably large influence on the surface morphology [17]. The laser energy density *E*_a_ can be expressed using Equation (3) [18],

(3) $$E_{a}\;=\;\frac{P}{V\;\times \;H}$$

where *P* is laser power, *V* is scanning speed, and *H* is the line spacing/hatch spacing between parallel scanning lines.

**Figure 12:**
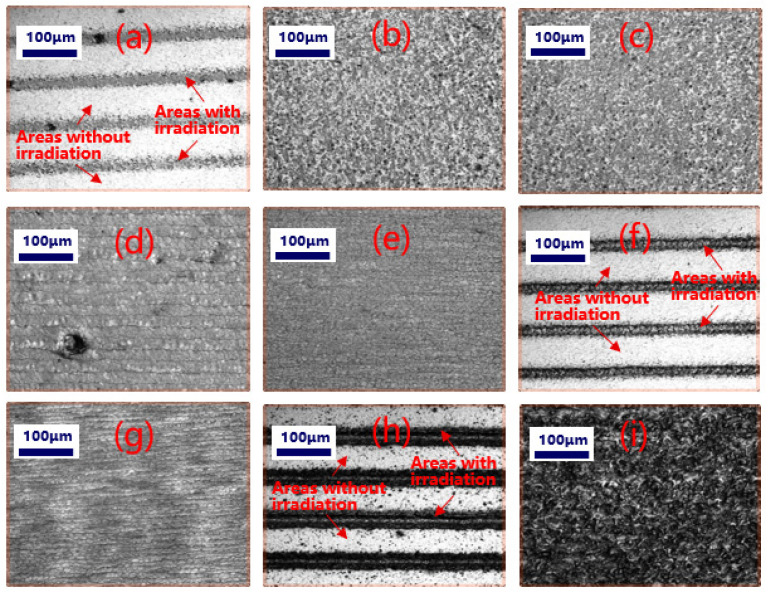
Surface roughness after laser surface modification: (**a**) Ra = 58 nm; (**b**) Ra = 68 nm; (**c**) Ra = 64 nm; (**d**) Ra = 149 nm; (**e**) Ra = 44 nm; (**f**) Ra = 256 nm; (**g**) Ra = 177 nm; (**h**) Ra = 144 nm; (**i**) Ra = 137 nm.

In experiments, the effects of *P*, *V* and *H* were investigated using the orthogonal experimental design method as shown in Table 2. According to the above parameters, the mirror surface was divided into 9 regions of 5 mm × 5 mm. After irradiation, the surface was investigated using a laser scanning confocal microscope (LSCM). The results of each region are shown in Figure 12. 

As shown in Figure 12a,f,h, the workpiece surface could not be completely modified. The energy distribution of the laser beam is in the shape of a bell curve (Gaussian shape) with a spot diameter of about 80 μm, while the line spacing was set to 100 μm. Therefore, the overlap of the laser beam in scanning was insufficient.

As shown in Figure 12b–d,i, the surfaces appeared to be pitted and flocculent. The metallographic structure was not yet uniform and refined due to insufficient energy density. 

As shown in Figure 12e,g, the workpiece surface became uniform. The surface roughness of (e) was better than (g), indicating that the parameters in line (e) in Table 2 were the most effective, where *P* = 10 W, *V* = 500 mm/s, *H* = 1 μm, *E*_a_ = 20 J/mm^2^.

After laser surface modification, 10 cycles of the cyclic heating and cooling were carried out to eliminate the possible surface residual stress introduced by laser irradiation. In every cycle, the temperature ranged from −50 °C to 120 °C under a heating or cooling rate of 2 °C per minute. 

### 3.4. Finish Machining of Mirror Surfaces

**Figure 13:**
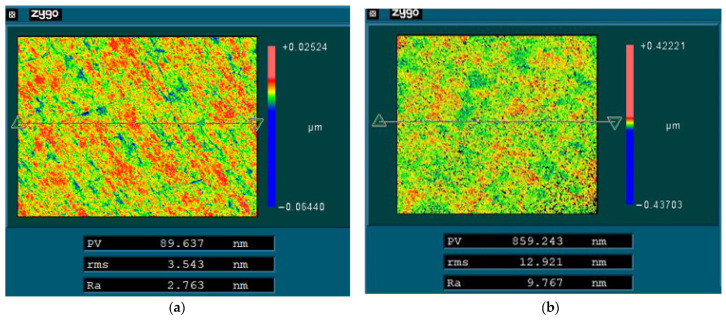
The roughness of SPDT surfaces: (**a**) with laser modification; (**b**) without laser modification.

**Figure 14:**
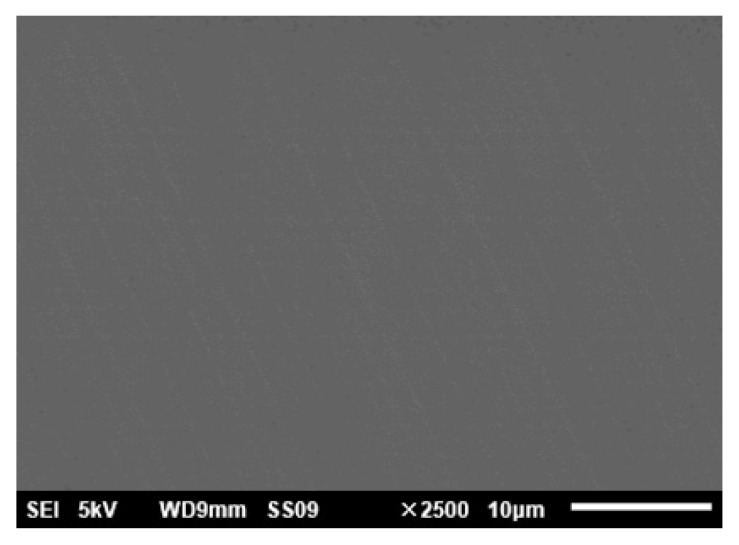
SEM micrograph of the SPDT surface after laser modification.

**Figure 15:**
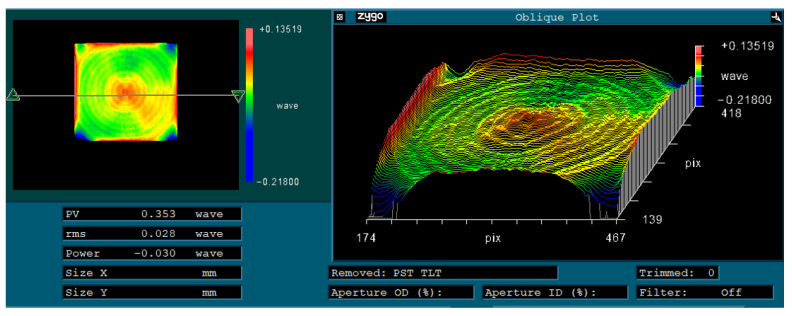
Shape accuracy of the SPDT surface after laser modification.

Finally, single-point diamond turning (SPDT) was used to finish the modified surface and the unmodified mirror surface, respectively. The parameters are listed in Table 3. The surfaces after SPDT were tested and the results are shown in Figure 13. The surface roughness of modified surface (Ra 2.8 nm) was significantly better than that of the unmodified (Ra 9.8 nm) surface. In order to further verify the effect of laser modification, the mirror surface with modification after finishing was investigated by SEM. The result is shown in Figure 14. Compared with Figure 11, the mirror surface with laser modification after finishing had a uniform metallographic structure without obvious unmolten particles and microscopic pores. The above results prove that laser surface modification is an effective means for the improvement of surface quality. The surface shape accuracy after SPDT was also tested using a Zygo interferometer. The result is shown in Figure 15. The PV and RMS value of the mirror surface were 0.353 λ and 0.028 λ (λ = 632.8 nm), respectively.

In terms of surface roughness and shape accuracy, the mirror surface fabricated in this paper can fully meet the application requirements of near infrared or even visible light optics. Future research could investigate the distribution of residual stress and the transformation of the metallographic structure after surface laser modification.

## 4. Conclusions

In this study, we designed and fabricated extremely lightweight truss-structured metal mirrors. The following conclusions were drawn:

1. Truss-structured mirrors show a better structural stability than that of conventional mirrors;
2. A very high weight reduction of up to 85% with nearly the same stiffness as traditional mirrors could be obtained;
3. By hot isostatic pressing, the porosity of AM mirror blanks was reduced. Through the aging treatment, the residual stress in the mirror blank after semi-finish machining was also eliminated;
4. After laser modification, the mirror surface had a uniform metallographic structure without obvious unmolten particles and microscopic pores.
5. After finishing, the surface shape accuracy was 0.353 λ (PV) and 0.028 λ (RMS) (λ = 632.8 nm). The surface roughness (Ra) of the mirrors was better than 3 nm for a bare metal mirror without NiP coating.
6. The truss-structured mirrors had strong temperature stability, due to the complete elimination of the bimetallic effect.

## Figures and Tables

**Table 1:** Main parameters used in design of the truss-structured mirrors.

Density/(g/cm^3^)	*t*/mm	Young’s Modulus/GPa	Poisson’s Ratio	Coefficient of Thermal Expansion/°C	*P*/Pa
2.7	2	70	0.3	21 ×10^−6^	52.3

**Table 2:** The orthogonal experimental design.

Regions *	Power (W)	Scanning Speed (mm/s)	Line Spacing (μm)
(a)	5	250	100
(b)	5	500	10
(c)	5	1000	1
(d)	10	250	10
(e)	10	500	1
(f)	10	1000	100
(g)	15	250	1
(h)	15	500	100
(i)	15	1000	10

*: Items in Coloum Regions are corresponding to subfigures in Figure 12.

**Table 3:** Cutting parameters in SPDT.

Cutting Tool	Single-Crystal Diamond Tool
Tool nose radius	1 mm
Rake angle	0°
Relief angle	10°
Feed rate	2 μm/rev
Speed of the spindle	1000 rev/min
Depth of cut	2 μm

## Data Availability

Not applicable.
